# Crystal structure of 4-(4-meth­oxy­phen­oxy)benzaldehyde

**DOI:** 10.1107/S2056989015022707

**Published:** 2015-12-06

**Authors:** Andreas Schäfer, Ljuba Iovkova-Berends, Stefan Gilke, Paul Kossmann, Hans Preut, Martin Hiersemann

**Affiliations:** a Fakultät Chemie und Chemische Biologie, Technische Universität Dortmund, Otto-Hahn-Strasse 6, 44221 Dortmund, Germany

**Keywords:** crystal structure, nucleophilic aromatic substitution, benzaldehyde

## Abstract

The title compound, C_14_H_12_O_3_, was synthesized *via* the nucleophilic addition of 4-meth­oxy­phenol to 4-fluoro­benzaldehyde. The dihedral angle between the least-squares planes of the benzene rings is 71.52 (3)° and the C—O—C angle at the central O atom is 118.82 (8)°. In the crystal, weak C—H⋯O hydrogen bonds link the molecules to generate supra­molecular layers in the *bc* plane. The layers are linked by weak C—H⋯π inter­actions.

## Related literature   

For the synthesis of 4-(4-meth­oxy­phen­oxy)benzaldehyde in an undergraduate laboratory course, see: Taber & Brannick (2015 ▸). For the synthesis of 4-aryl­oxybenzaldehydes and aceto­phenones, see: Yeager & Schissel (1991 ▸).
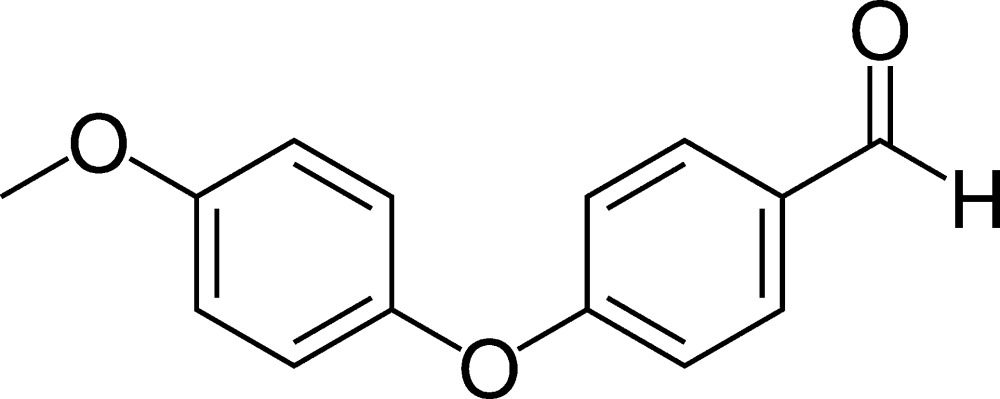


## Experimental   

### Crystal data  

C_14_H_12_O_3_
*M* *_r_* = 228.24
Monoclinic, 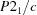

*a* = 12.1297 (7) Å
*b* = 7.6581 (4) Å
*c* = 12.3577 (7) Å
β = 103.769 (6)°
*V* = 1114.92 (11) Å^3^
*Z* = 4
Mo *K*α radiation
μ = 0.10 mm^−1^
*T* = 173 K
0.56 × 0.40 × 0.30 mm

### Data collection  

Oxford Diffraction Xcalibur2 CCD diffractometer
Absorption correction: multi-scan (*CrysAlis RED*; Oxford Diffraction, 2008 ▸) *T*_min_ = 0.808, *T*_max_ = 1.000
10049 measured reflections
2967 independent reflections
2551 reflections with *I* > 2σ(*I*)
*R*_int_ = 0.023

### Refinement  

*R*[*F* ^2^ > 2σ(*F* ^2^)] = 0.040
*wR*(*F* ^2^) = 0.112
*S* = 1.04
2967 reflections
155 parameters
H-atom parameters constrained
Δρ_max_ = 0.28 e Å^−3^
Δρ_min_ = −0.22 e Å^−3^

### 

Data collection: *CrysAlis PRO* (Oxford Diffraction, 2008 ▸); cell refinement: *CrysAlis PRO*; data reduction: *CrysAlis PRO*; program(s) used to solve structure: *SHELXS2014* (Sheldrick, 2008 ▸); program(s) used to refine structure: *SHELXL2013* (Sheldrick, 2015 ▸); molecular graphics: *SHELXP2014* (Sheldrick, 2008 ▸); software used to prepare material for publication: *SHELXL2013* and *PLATON* (Spek, 2009 ▸).

## Supplementary Material

Crystal structure: contains datablock(s) I, 3352b. DOI: 10.1107/S2056989015022707/tk5411sup1.cif

Structure factors: contains datablock(s) I. DOI: 10.1107/S2056989015022707/tk5411Isup2.hkl

Supporting information file. DOI: 10.1107/S2056989015022707/tk5411Isup3.cml

**Fig. 1.**
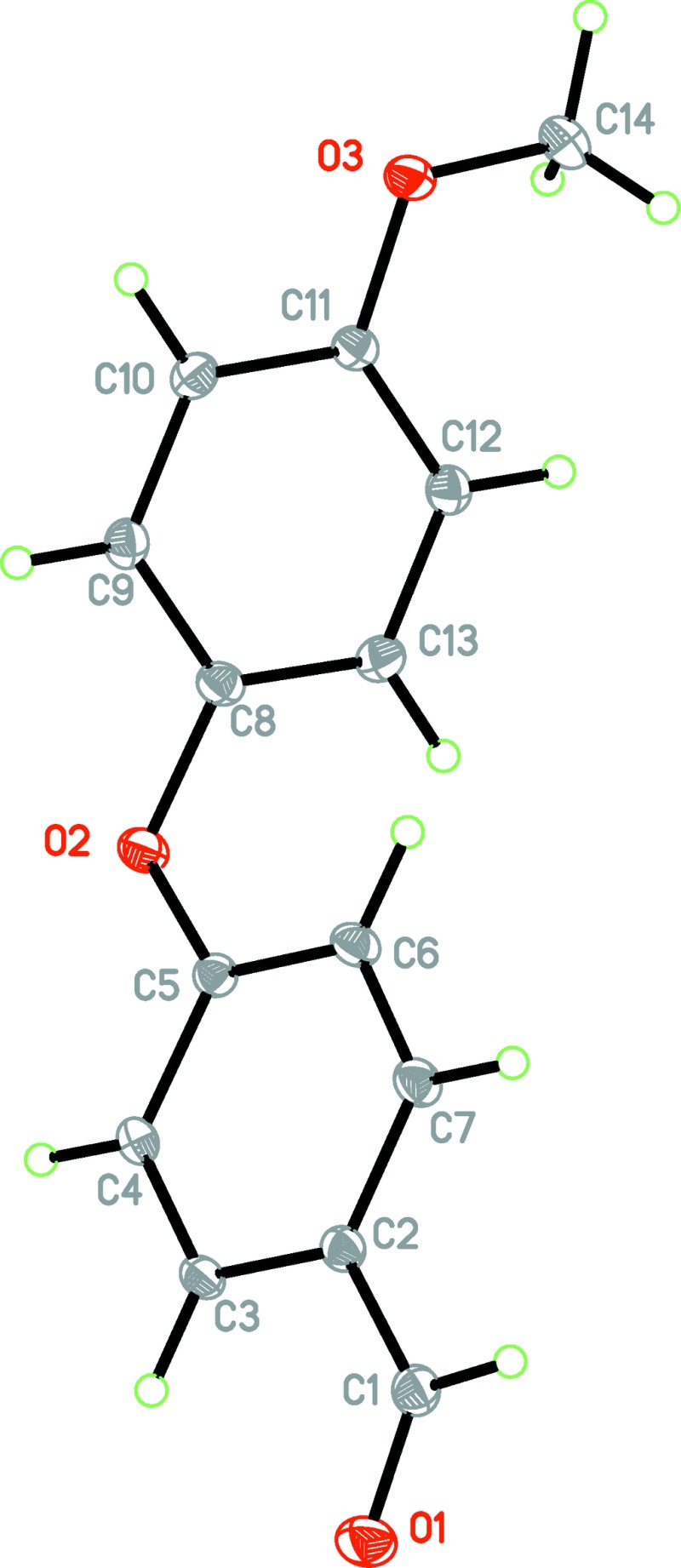
The molecular structure of the title compound, showing the labelling of all non-H atoms. Displacement ellipsoids are shown at the 50% probability level.

. DOI: 10.1107/S2056989015022707/tk5411fig1.tif
The mol­ecular structure of the title compound, showing the labelling of all non-H atoms. Displacement ellipsoids are shown at the 50% probability level.

CCDC reference: 1439095

Additional supporting information:  crystallographic information; 3D view; checkCIF report

## Figures and Tables

**Table 1:** Hydrogen-bond geometry (Å, °) *Cg*1 and *Cg*2 are the centroids of the C2–C7 and C8–C13 rings, respectively.

*D*—H⋯*A*	*D*—H	H⋯*A*	*D*⋯*A*	*D*—H⋯*A*
C13—H13*A*⋯O1^i^	0.95	2.58	3.5129 (14)	167
C7—H7*A*⋯O1^ii^	0.95	2.56	3.2500 (14)	130
C1—H1*A*⋯*Cg*1^iii^	0.95	2.73	3.5453 (12)	145
C10—H10*A*⋯*Cg*2^iv^	0.95	2.88	3.7465 (12)	152

Symmetry codes: (i) 
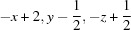
; (ii) 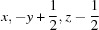; (iii) 
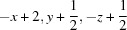
; (iv) 
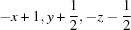
.

## supplementary crystallographic information

### S1. Comment

As part of an evaluation of single step experiments for an undergraduate laboratory course, we came across a literature protocol that describes the preparation of crystalline 4-aryloxybenzaldehyde by nucleophilic aromatic substitution (Taber & Brannick, 2015). The reaction of 4-fluorobenzaldehyde (II) with 4-methoxyphenol (III) in the presence of potassium carbonate in dimethyl sulfoxide provided 4-(4-methoxyphenoxy)benzaldehyde (I) as large pale yellow crystals. The recrystallization of a small amount of (I) from *n*-heptane provided clear colourless crystals, suitable for X-ray analysis. In our hands, the literature protocol failed to deliver precipitated crude product upon dilution of the reaction mixture with water and subsequent drying on filter paper. Our modified protocol is characterized by a general aqueous work-up procedure, including extraction with brine for removal of dimethyl sulfoxide.

### S2. Experimental

In a glass test tube (160x16 mm) 4-fluorobenzaldehyde (II) (C_7_H_5_FO, *M* =124.11 g/mol, 250 mg, 2.01 mmol, 1 eq), 4-methoxyphenol (III) (C_7_H_8_O_2_, *M* = 124.14 g/mol, 250 mg, 2.01 mmol, 1 eq) and potassium carbonate (K_2_CO_3_, *M* = 138.20 g/mol, 550 mg, 3.98 mmol, 2 eq) were suspended in dimethyl sulfoxide (2 ml, 1 ml/mmol). The reaction mixture was heated to 413 K and stirred at this temperature for 45 min. After consumption of the starting materials, the oil bath was removed and the suspension was cooled to room temperature. The reaction mixture was diluted with water (6 ml, 3 ml/mmol) and stirred at ambient temperature for 30 min. The resulting suspension was tranferred into a separatory funnel with water and then extracted with ethyl acetate (3x). The combined organic phases were extracted with saturated aqueous sodium chloride solution (5x) and dried over MgSO_4_. After removal of the solvents under reduced pressure, the light brown viscous oil was dissolved in dichloromethane (2 ml) and transferred into a wide-necked flask. The solution was diluted with *n*-heptane (1 ml) and the solvent was allowed to evaporate over three days. Crystals slowly form and grow, coating the sides of the flask. The large pale yellow crystals were washed with *n*-heptane (1 ml) and dried *in vacuo* to deliver 4-(4-methoxyphenoxy)benzaldehyde (I) (C_14_H_12_O_3_, *M* = 228.25 g/mol, 440 mg, 1.93 mmol, 96%). Recrystallization of a small amount of (I) from *n*-heptane by slow evaporation over one week provided clear colourless crystals. *R*_f_ 0.48 (cyclohexane/ethyl acetate 5/1); m.p. 323–325 K (*n*-heptane) [m.p. 332.5–333.5 K (*n*-hexane) (Yeager & Schissel, 1991)]; ^1^H NMR (CDCl_3_, 500 MHz) δ 3.83 (s, 3H), 6.92–6.95 (m, 2H), 6.99–7.05 (m, 4H), 7.81–7.83 (m, 2H), 9.90 (s, 1H); ^13^C NMR (CDCl_3_, 126 MHz) δ 55.8 (CH_3_), 115.3 (CH), 116.9 (CH), 122.0 (CH), 131.0 (C), 132.1 (CH), 148.3 (C), 157.0 (C), 164.2 (C), 190.9 (CH); IR ν 3005 (w), 2965 (w), 2835 (w), 2745 (w), 1680 (*s*), 1595 (*m*), 1575 (*s*), 1495 (*s*), 1440 (*m*), 1230 (*s*), 1195 (*s*), 1150 (*s*), 1100 (*m*), 1085 (*s*), 875 (*m*), 845 (*m*), 830 (*s*), 785 (*s*), 745 (*m*), 565 (*m*), 525 (*m*), 510 (*s*).

### S3. Refinement

H-atoms attached to C, except those in CH_3_, were placed in calculated positions (C—H = 0.95 Å and *U*_iso_(H) = 1.2 *U*_eq_(C)). CH_3_ hydrogen atoms, which were taken from a Fourier map, were allowed to rotate but not to tip (C—H = 0.98 Å and *U*_iso_(H) = 1.5 *U*_eq_(C)).

### Crystal data

**Table:**

C_14_H_12_O_3_	*D*_x_ = 1.360 Mg m^−^^3^
*M**_r_* = 228.24	Melting point = 323–325 K
Monoclinic, *P*2_1_/*c*	Mo *K*α radiation, λ = 0.71073 Å
*a* = 12.1297 (7) Å	Cell parameters from 10396 reflections
*b* = 7.6581 (4) Å	θ = 3.2–31.0°
*c* = 12.3577 (7) Å	µ = 0.10 mm^−^^1^
β = 103.769 (6)°	*T* = 173 K
*V* = 1114.92 (11) Å^3^	Block, colourless
*Z* = 4	0.56 × 0.40 × 0.30 mm
*F*(000) = 480	

### Data collection

**Table:**

Oxford Diffraction Xcalibur2 CCD diffractometer	2967 independent reflections
Radiation source: fine-focus sealed tube	2551 reflections with *I* > 2σ(*I*)
Detector resolution: 16.0560 pixels mm^-1^	*R*_int_ = 0.023
ω und ψ scan	θ_max_ = 29.0°, θ_min_ = 3.2°
Absorption correction: multi-scan (*CrysAlis RED*; Oxford Diffraction, 2008)	*h* = −16→16
*T*_min_ = 0.808, *T*_max_ = 1.000	*k* = −10→10
10049 measured reflections	*l* = −16→14

### Refinement

**Table:**

Refinement on *F*^2^	Primary atom site location: structure-invariant direct methods
Least-squares matrix: full	Secondary atom site location: difference Fourier map
*R*[*F*^2^ > 2σ(*F*^2^)] = 0.040	Hydrogen site location: inferred from neighbouring sites
*wR*(*F*^2^) = 0.112	H-atom parameters constrained
*S* = 1.04	*w* = 1/[σ^2^(*F*_o_^2^) + (0.0604*P*)^2^ + 0.2707*P*] where *P* = (*F*_o_^2^ + 2*F*_c_^2^)/3
2967 reflections	(Δ/σ)_max_ < 0.001
155 parameters	Δρ_max_ = 0.28 e Å^−^^3^
0 restraints	Δρ_min_ = −0.22 e Å^−^^3^

### Special details

**Table:**

Geometry. All e.s.d.'s (except the e.s.d. in the dihedral angle between two l.s. planes) are estimated using the full covariance matrix. The cell e.s.d.'s are taken into account individually in the estimation of e.s.d.'s in distances, angles and torsion angles; correlations between e.s.d.'s in cell parameters are only used when they are defined by crystal symmetry. An approximate (isotropic) treatment of cell e.s.d.'s is used for estimating e.s.d.'s involving l.s. planes.
Refinement. Refinement of *F*^2^ against ALL reflections. The weighted *R*-factor *wR* and goodness of fit *S* are based on *F*^2^, conventional *R*-factors *R* are based on *F*, with *F* set to zero for negative *F*^2^. The threshold expression of *F*^2^ > σ(*F*^2^) is used only for calculating *R*-factors(gt) *etc*. and is not relevant to the choice of reflections for refinement. *R*-factors based on *F*^2^ are statistically about twice as large as those based on *F*, and *R*- factors based on ALL data will be even larger.

### Fractional atomic coordinates and isotropic or equivalent isotropic displacement parameters (Å^2^)

**Table:**

	*x*	*y*	*z*	*U*_iso_*/*U*_eq_	
O1	1.04659 (7)	0.20198 (13)	0.46684 (7)	0.0345 (2)	
C1	1.03112 (9)	0.22947 (15)	0.36759 (9)	0.0254 (2)	
H1A	1.0880	0.2933	0.3435	0.030*	
O2	0.65559 (6)	0.00429 (11)	0.03836 (6)	0.02476 (19)	
C2	0.93213 (9)	0.17254 (14)	0.28176 (8)	0.0207 (2)	
O3	0.60491 (7)	0.07872 (11)	−0.41611 (6)	0.02479 (19)	
C3	0.84285 (9)	0.07946 (14)	0.30868 (8)	0.0207 (2)	
H3A	0.8453	0.0524	0.3842	0.025*	
C4	0.75139 (9)	0.02708 (14)	0.22570 (8)	0.0204 (2)	
H4A	0.6902	−0.0343	0.2440	0.025*	
C5	0.74924 (8)	0.06494 (13)	0.11433 (8)	0.0191 (2)	
C6	0.83733 (9)	0.15660 (14)	0.08603 (8)	0.0225 (2)	
H6A	0.8354	0.1817	0.0103	0.027*	
C7	0.92811 (9)	0.21065 (14)	0.17041 (9)	0.0233 (2)	
H7A	0.9884	0.2745	0.1522	0.028*	
C8	0.65010 (9)	0.02527 (14)	−0.07551 (8)	0.0210 (2)	
C9	0.56903 (9)	0.13878 (14)	−0.13564 (9)	0.0221 (2)	
H9A	0.5228	0.2056	−0.0990	0.026*	
C10	0.55637 (9)	0.15361 (14)	−0.24972 (9)	0.0221 (2)	
H10A	0.5012	0.2310	−0.2916	0.027*	
C11	0.62441 (8)	0.05530 (13)	−0.30341 (8)	0.0198 (2)	
C12	0.70607 (9)	−0.05710 (14)	−0.24197 (9)	0.0228 (2)	
H12A	0.7531	−0.1232	−0.2781	0.027*	
C13	0.71864 (9)	−0.07229 (14)	−0.12726 (9)	0.0239 (2)	
H13A	0.7739	−0.1491	−0.0849	0.029*	
C14	0.66492 (10)	−0.03182 (16)	−0.47516 (9)	0.0280 (2)	
H14A	0.7466	−0.0111	−0.4488	0.042*	
H14B	0.6412	−0.0062	−0.5550	0.042*	
H14C	0.6482	−0.1542	−0.4622	0.042*	

### Atomic displacement parameters (Å^2^)

**Table:**

	*U*^11^	*U*^22^	*U*^33^	*U*^12^	*U*^13^	*U*^23^
O1	0.0325 (4)	0.0469 (5)	0.0221 (4)	−0.0060 (4)	0.0023 (3)	−0.0031 (4)
C1	0.0219 (5)	0.0302 (6)	0.0241 (5)	−0.0030 (4)	0.0057 (4)	−0.0041 (4)
O2	0.0225 (4)	0.0336 (4)	0.0178 (4)	−0.0075 (3)	0.0041 (3)	0.0004 (3)
C2	0.0216 (5)	0.0215 (5)	0.0194 (5)	0.0002 (4)	0.0058 (4)	−0.0017 (4)
O3	0.0275 (4)	0.0287 (4)	0.0177 (4)	0.0030 (3)	0.0047 (3)	−0.0011 (3)
C3	0.0238 (5)	0.0228 (5)	0.0168 (4)	0.0009 (4)	0.0073 (4)	0.0002 (4)
C4	0.0205 (5)	0.0217 (5)	0.0211 (5)	−0.0012 (4)	0.0088 (4)	0.0007 (4)
C5	0.0194 (5)	0.0192 (5)	0.0187 (5)	0.0004 (3)	0.0042 (4)	−0.0005 (4)
C6	0.0256 (5)	0.0241 (5)	0.0185 (5)	−0.0027 (4)	0.0067 (4)	0.0034 (4)
C7	0.0236 (5)	0.0254 (5)	0.0222 (5)	−0.0046 (4)	0.0079 (4)	0.0013 (4)
C8	0.0209 (5)	0.0241 (5)	0.0175 (4)	−0.0053 (4)	0.0037 (4)	−0.0010 (4)
C9	0.0195 (5)	0.0233 (5)	0.0238 (5)	−0.0010 (4)	0.0061 (4)	−0.0044 (4)
C10	0.0192 (5)	0.0229 (5)	0.0227 (5)	0.0013 (4)	0.0020 (4)	−0.0011 (4)
C11	0.0205 (5)	0.0203 (5)	0.0183 (4)	−0.0035 (4)	0.0040 (4)	−0.0016 (4)
C12	0.0228 (5)	0.0234 (5)	0.0233 (5)	0.0023 (4)	0.0074 (4)	−0.0010 (4)
C13	0.0224 (5)	0.0249 (5)	0.0235 (5)	0.0023 (4)	0.0036 (4)	0.0033 (4)
C14	0.0323 (6)	0.0309 (6)	0.0232 (5)	0.0002 (5)	0.0115 (4)	−0.0027 (4)

### Geometric parameters (Å, º)

**Table:**

O1—C1	1.2142 (14)	C6—H6A	0.9500
C1—C2	1.4664 (14)	C7—H7A	0.9500
C1—H1A	0.9500	C8—C13	1.3826 (15)
O2—C5	1.3711 (12)	C8—C9	1.3881 (15)
O2—C8	1.4021 (12)	C9—C10	1.3861 (14)
C2—C7	1.3962 (14)	C9—H9A	0.9500
C2—C3	1.4010 (14)	C10—C11	1.3959 (14)
O3—C11	1.3678 (12)	C10—H10A	0.9500
O3—C14	1.4254 (13)	C11—C12	1.3931 (14)
C3—C4	1.3791 (14)	C12—C13	1.3940 (14)
C3—H3A	0.9500	C12—H12A	0.9500
C4—C5	1.4006 (13)	C13—H13A	0.9500
C4—H4A	0.9500	C14—H14A	0.9800
C5—C6	1.3909 (14)	C14—H14B	0.9800
C6—C7	1.3873 (14)	C14—H14C	0.9800
			
O1—C1—C2	125.60 (10)	C13—C8—O2	120.81 (9)
O1—C1—H1A	117.2	C9—C8—O2	117.86 (9)
C2—C1—H1A	117.2	C10—C9—C8	119.25 (10)
C5—O2—C8	118.82 (8)	C10—C9—H9A	120.4
C7—C2—C3	119.52 (9)	C8—C9—H9A	120.4
C7—C2—C1	118.77 (10)	C9—C10—C11	120.29 (9)
C3—C2—C1	121.71 (9)	C9—C10—H10A	119.9
C11—O3—C14	117.25 (8)	C11—C10—H10A	119.9
C4—C3—C2	120.13 (9)	O3—C11—C12	124.34 (9)
C4—C3—H3A	119.9	O3—C11—C10	115.76 (9)
C2—C3—H3A	119.9	C12—C11—C10	119.89 (9)
C3—C4—C5	119.60 (9)	C11—C12—C13	119.82 (10)
C3—C4—H4A	120.2	C11—C12—H12A	120.1
C5—C4—H4A	120.2	C13—C12—H12A	120.1
O2—C5—C6	124.03 (9)	C8—C13—C12	119.54 (9)
O2—C5—C4	114.91 (9)	C8—C13—H13A	120.2
C6—C5—C4	121.05 (9)	C12—C13—H13A	120.2
C7—C6—C5	118.78 (9)	O3—C14—H14A	109.5
C7—C6—H6A	120.6	O3—C14—H14B	109.5
C5—C6—H6A	120.6	H14A—C14—H14B	109.5
C6—C7—C2	120.91 (10)	O3—C14—H14C	109.5
C6—C7—H7A	119.5	H14A—C14—H14C	109.5
C2—C7—H7A	119.5	H14B—C14—H14C	109.5
C13—C8—C9	121.21 (9)		
			
O1—C1—C2—C7	−178.37 (11)	C5—O2—C8—C13	71.52 (13)
O1—C1—C2—C3	0.79 (18)	C5—O2—C8—C9	−112.38 (11)
C7—C2—C3—C4	−0.45 (16)	C13—C8—C9—C10	0.34 (15)
C1—C2—C3—C4	−179.60 (10)	O2—C8—C9—C10	−175.74 (9)
C2—C3—C4—C5	1.06 (16)	C8—C9—C10—C11	0.04 (15)
C8—O2—C5—C6	4.13 (15)	C14—O3—C11—C12	6.25 (15)
C8—O2—C5—C4	−175.51 (9)	C14—O3—C11—C10	−174.01 (9)
C3—C4—C5—O2	178.84 (9)	C9—C10—C11—O3	179.69 (9)
C3—C4—C5—C6	−0.82 (16)	C9—C10—C11—C12	−0.56 (15)
O2—C5—C6—C7	−179.68 (10)	O3—C11—C12—C13	−179.58 (9)
C4—C5—C6—C7	−0.06 (16)	C10—C11—C12—C13	0.69 (15)
C5—C6—C7—C2	0.69 (16)	C9—C8—C13—C12	−0.21 (16)
C3—C2—C7—C6	−0.44 (16)	O2—C8—C13—C12	175.76 (9)
C1—C2—C7—C6	178.74 (10)	C11—C12—C13—C8	−0.31 (16)

### Hydrogen-bond geometry (Å, º)

*Cg*1 and *Cg*2 are the centroids of the C2–C7 and C8–C13 rings, respectively.

**Table:**

*D*—H···*A*	*D*—H	H···*A*	*D*···*A*	*D*—H···*A*
C13—H13*A*···O1^i^	0.95	2.58	3.5129 (14)	167
C7—H7*A*···O1^ii^	0.95	2.56	3.2500 (14)	130
C1—H1*A*···*Cg*1^iii^	0.95	2.73	3.5453 (12)	145
C10—H10*A*···*Cg*2^iv^	0.95	2.88	3.7465 (12)	152

Symmetry codes: (i) −*x*+2, *y*−1/2, −*z*+1/2; (ii) *x*, −*y*+1/2, *z*−1/2; (iii) −*x*+2, *y*+1/2, −*z*+1/2; (iv) −*x*+1, *y*+1/2, −*z*−1/2.
