# Supplementary figures and images for: Crystal structure of 4-(4-meth­oxy­phen­oxy)benzaldehyde
Source: Acta Crystallogr E Crystallogr Commun. 2015 Dec 6;71(Pt 12):o1021. doi: 10.1107/S2056989015022707 (PMC4719957; doi:10.1107/S2056989015022707)

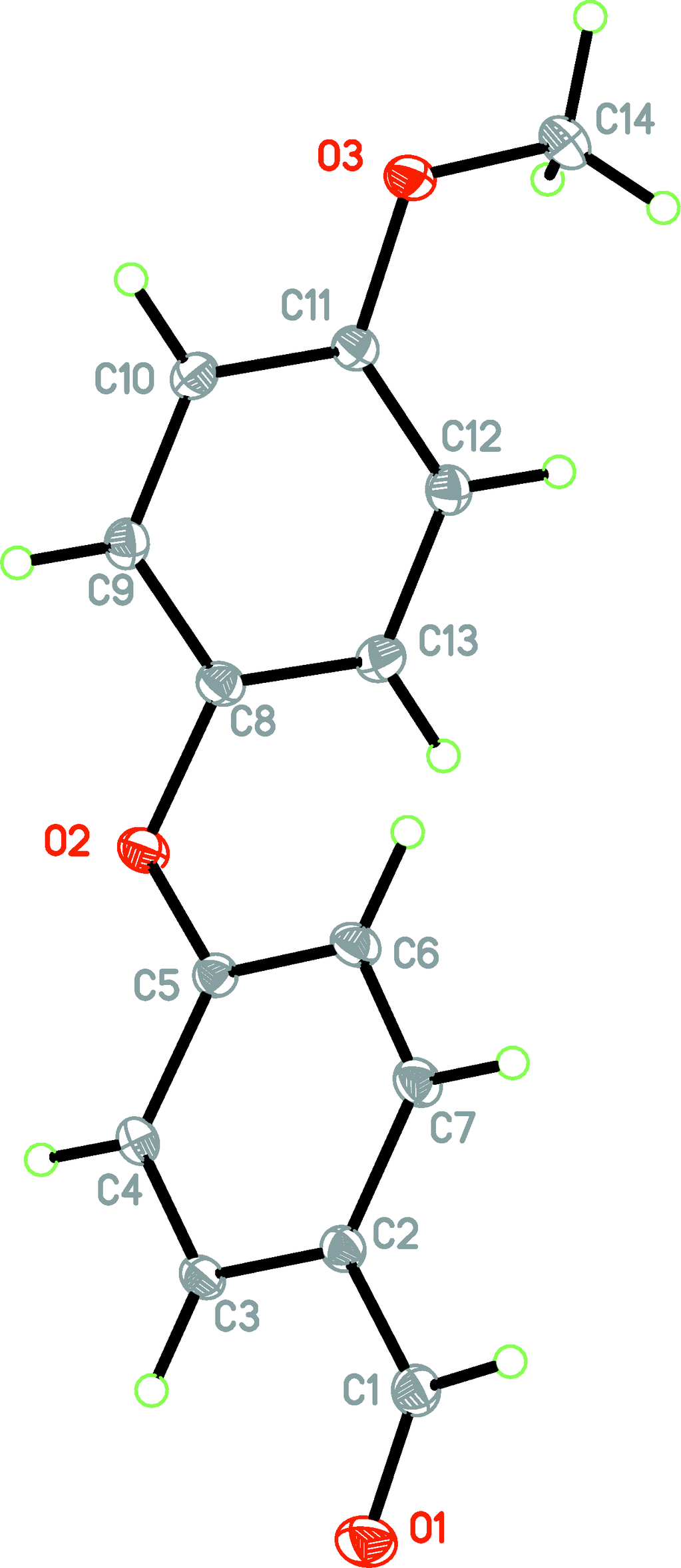

Supplement: Supplementary file 4 [file e-71-o1021-fig1.tif]
